# Somatic *GPR101* Duplication Causing X-Linked Acrogigantism (XLAG)—Diagnosis and Management

**DOI:** 10.1210/jc.2015-4366

**Published:** 2016-03-16

**Authors:** Celia Rodd, Maude Millette, Donato Iacovazzo, Craig E. Stiles, Sayka Barry, Jane Evanson, Steffen Albrecht, Richard Caswell, Benjamin Bunce, Sian Jose, Jacqueline Trouillas, Federico Roncaroli, Julian Sampson, Sian Ellard, Márta Korbonits

**Affiliations:** Pediatrics and Child Health (C.R.), University of Manitoba, Winnipeg MB R3E 0Z2, Canada; Department of Pediatrics (M.M.), Centre mère-enfant Soleil, Centre Hospitalier de l'Université de Quebec, QC G1V 4G2, Canada; Endocrinology (D.I., C.E.S., S.B., J.E., M.K.), Barts and the London School of Medicine, Queen Mary University of London, London EC1M 6BQ, United Kingdom; Department of Pathology (S.A.), McGill University, Montreal QC H4A 2J1, Canada; Molecular Genetics (R.C., B.B., S.E.), University of Exeter, Exeter EX4 4SB, United Kingdom; Institute of Medical Genetics (S.J., J.S.), School of Medicine, Cardiff University, Cardiff CF10 3XQ, United Kingdom; Centre de Pathologie Est (J.T.), Hospices Civils de Lyon, University of Lyon, 69622 Lyon, France; and Neuropathology (F.R.), University of Manchester, Manchester M13 9PL, United Kingdom

## Abstract

**Context:**

Recent reports have proposed that sporadic or familial germline Xq26.3 microduplications involving the *GPR101* gene are associated with early-onset X-linked acrogigantism (XLAG) with a female preponderance.

**Case Description:**

A 4-year-old boy presented with rapid growth over the previous 2 years. He complained of sporadic headaches and had coarse facial features. His height Z-score was +4.89, and weight Z-score was +5.57. Laboratory testing revealed elevated serum prolactin (185 μg/L; normal, <18 μg/L), IGF-1 (745 μg/L; normal, 64–369 μg/L), and fasting GH > 35.0 μg/L. Magnetic resonance imaging demonstrated a homogenous bulky pituitary gland (18 × 15 × 13 mm) without obvious adenoma. A pituitary biopsy showed hyperplastic pituitary tissue with enlarged cords of GH and prolactin cells. Germline *PRKAR1A*, *MEN1*, *AIP*, *DICER1*, *CDKN1B*, and somatic *GNAS* mutations were negative. Medical management was challenging until institution of continuous sc infusion of short-acting octreotide combined with sc pegvisomant and oral cabergoline. The patient remains well controlled with minimal side effects 7 years after presentation. His phenotype suggested XLAG, but his peripheral leukocyte-, saliva-, and buccal cell-derived DNA tested negative for microduplication in Xq26.3 or *GPR101*. However, DNA isolated from the pituitary tissue and forearm skin showed duplicated dosage of *GPR101*, suggesting that he is mosaic for this genetic abnormality.

**Conclusions:**

Our patient is the first to be described with somatic microduplication leading to typical XLAG phenotype. This patient demonstrates that a negative test for Xq26.3 microduplication or *GPR101* duplication on peripheral blood DNA does not exclude the diagnosis of XLAG because it can result from a mosaic mutation affecting the pituitary.

## Abstract (precis)

XLAG can result from somatic mutation resulting in mosaicism. Combination of sc octreotide infusion, pegvisomant and cabergoline resulted in well controlled IGF-1 and height velocity.

X-linked acrogigantism (XLAG) is a recently described clinical syndrome of early-onset gigantism with typical onset in the first few months of life and more often affecting females (1–3). The disease is associated with microduplications at Xq26.3 involving the *GPR101* gene, which encodes a G protein-coupled orphan receptor. Most of the patients present with a GH and prolactin (PRL)-secreting pituitary adenoma or less commonly GH and PRL cell hyperplasia. The vast majority of the previously described patients required extensive anterior pituitary resection or radiotherapy, resulting in hypopituitarism to control exuberant hormone secretion and growth velocity, because medical management with somatostatin analogs has not been sufficient (3).

## Case Report and Methods

**Figure 1.**
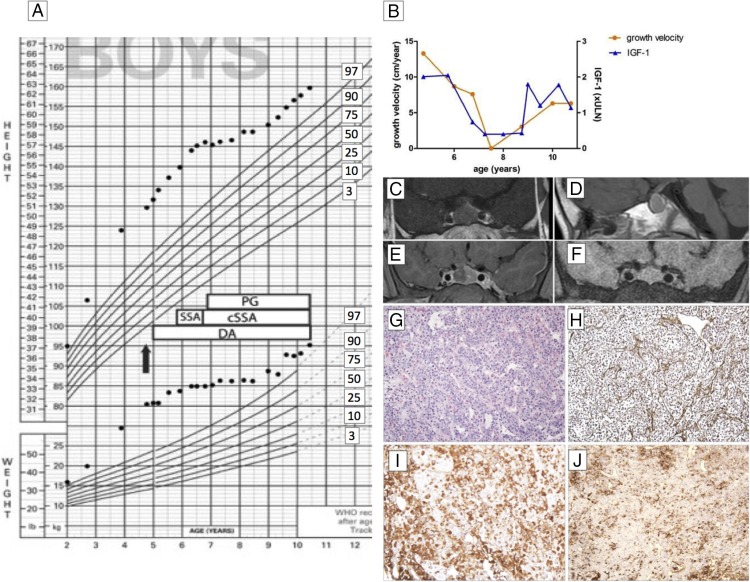
A, Growth chart before and after surgical and medical interventions. Treatment strategies are indicated as follows: arrow, diagnosis and surgery; DA, dopamine agonist (initially bromocriptine then followed by cabergoline); SSA, somatostatin analog (initially Sandostatin LAR and Somatuline Autogel, then continuous sc infusion [cSSA] administered at 120–320 μg/d dose); and PG, pegvisomant dose, 30–105 mg/wk. B, Growth velocity in centimeters per year (left y-axis, circles) and IGF-1 concentrations (right y-axis, triangles; fold above 95% upper reference limit for age). C—F, Magnetic resonance imaging of the pituitary gland, coronal (C) and sagittal (D) view before surgery, and 5 weeks (E) and 24 months (F) after surgery. G and H, Histological examination of the pituitary tissue demonstrates normal architecture of the gland with preservation of the reticulin network but markedly enlarged cell cords, suggesting diffuse hyperplasia: hematoxylin-eosin staining, ×10 (G); and Gömöri's reticulin stain, ×10 (H). I and J, The gland shows an increase in GH-positive (immunoperoxidase, ×10; I) and PRL-positive cells (immunoperoxidase, ×10; J).

A 4-year and 8-month-old Caucasian boy presented to the Montreal Children's Hospital with rapid growth over the preceding 2 years. Born at term with a weight of 4.7 kg (>95th percentile), he had grown steadily along the 90th percentile for length up to 24 months of age and was above the median for weight and head circumference. He had undergone a tonsillectomy and adenoidectomy at the age of 3 years and 10 months. He complained of intermittent headaches over the preceding 8 months, was otherwise asymptomatic, and took no medications. His family history was unremarkable for any endocrinopathies; his midparental height was 174.5 cm. On examination, he appeared older than his stated age, was prepubertal, and had coarse facial features, which had developed over the previous 3 years. His height was 129.7 cm (Z = +4.89), and his weight was 35.5 kg (Z = +5.57) (WHO Growth Charts for Canada; Ref. 4) (Figure 1A).

Laboratory investigations revealed elevated serum concentrations of PRL (185 μg/L; normal, <18 μg/L [8043 pmol/L; normal, <788 pmol/L]; Access II; Beckman Coulter), IGF-1 (74.5 μg/L; normal for age, 6.4–36.9 μg/L [9.78 nmol/L, 0.8–4.8]; Immulite), and a fasting GH of > 35.0 μg/L (Access II; Beckman Coulter) (Figure 1B). GH suppressed only to 29.0 μg/L after standard oral glucose load (normal, <1.0). His clinical and biochemical diagnosis was pituitary gigantism with hyperprolactinemia. Magnetic resonance imaging demonstrated a homogeneous sellar mass measuring 18 × 15 × 13 mm with mild suprasellar extension without optic chiasm or cavernous sinus invasion (Figure 1, C and D). Surgical debulking was performed via transfrontal craniotomy. Histological examination of the tissue confirmed the absence of adenoma and demonstrated a hyperplastic pituitary with enlarged cords of GH and PRL cells (Figure 1, G–J). Cells with ultrastructural features resembling mammosomatotrophs were also identified. Genetic analysis was negative for germline *AIP*, *PRKAR1A*, *DICER1*, *MEN1*, and *CDKN1B* mutations and for *GNAS* mutations in both leukocyte and pituitary tissue-derived DNA. Plasma GHRH (Inter Science Institute) levels were normal on two separate samples. His bone age was 5 years.

Postoperatively, his hormone levels remained elevated; the hyperplastic tissue re-expanded rapidly in the fossa (Figure 1, E and F), and his growth continued at an accelerated pace (Figure 1, A and B). His management was challenging. Dopamine agonists proved unsuccessful in suppressing GH hypersecretion; bromocriptine was initiated for the first 3 months but was later replaced with cabergoline (0.25–0.5 mg/wk) to control PRL levels. Long-acting somatostatin analog therapy induced a slight drop in GH but not in IGF-1 levels. Because the patient refused monthly injections (initially Sandostatin LAR and then Somatuline Autogel), after about 9 months, treatment was switched to continuous sc infusion of short-acting octreotide (5) at doses ranging from 120–320 μg/d. The combination of somatostatin analog and cabergoline was insufficient to normalize GH or IGF-1 levels. The addition of the GH receptor antagonist pegvisomant (30–105 mg/wk) normalized IGF-1 and induced a plateau in his growth; the pituitary volume remained stable with this treatment combination (Figure 1F). Around the age of 7.5 years, the parents wished to reduce the dose of pegvisomant and short-acting octreotide therapy to allow controlled growth. They felt that he was destined to be a tall boy; thereafter his growth followed parallel to the 97th height percentile (Figure 1A), and his IGF-1 remained within the normal range.

Currently, at the age of 11 years, he remains well; his growth velocity is normal. He is on oral cabergoline 125 μg thrice weekly, short-acting sc octreotide 222 μg/24 h, and sc pegvisomant 20 mg twice weekly.

**Figure 2.**
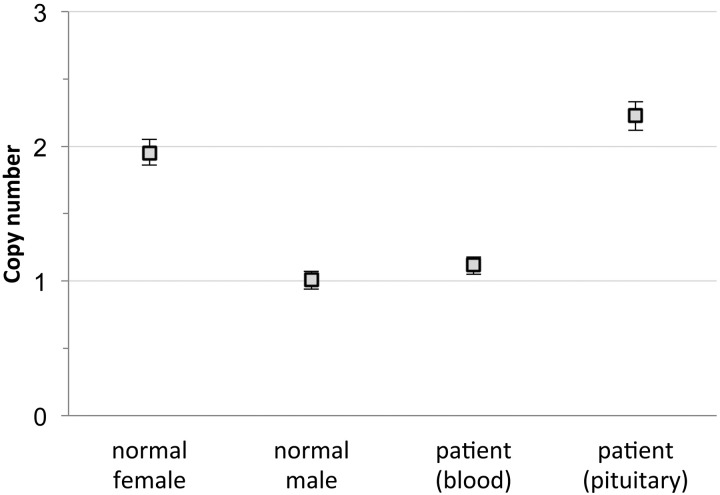
*GPR101* copy number as measured with copy number variation droplet digital PCR (assay id Hs01818174_cn, Life Technologies), showing two copies of *GPR101* for normal female control, one copy for normal male control, one copy for the patient's blood-derived DNA, and two copies for the patient's pituitary tissue-derived DNA. Vertical bars show 95% Poisson confidence limits.

After the recent description of germline Xq26.3 microduplication in young children with acrogigantism (1), likely due to the duplicated *GPR101* gene within this region, we tested the patient's leukocyte-, saliva-, and buccal cell-derived DNA using a comparative genomic hybridization array (BlueGnome CytoChip ISCA 8 × 60k v2.0; Illumina) and copy number variation droplet digital PCR for *GPR101* (Taqman assays Hs01818174_cn and Hs01730605_cn; Life Technologies) without evidence of Xq26.3 microduplications or *GPR101* duplication, although his clinical phenotype was identical to the previously published patients. We therefore tested DNA isolated from the hyperplastic pituitary tissue and found duplicated dosage of *GPR101* (Figure 2). DNA extracted from a forearm skin biopsy also showed the microduplication, suggesting that he is mosaic for this mutation.

Ethics approval was granted from the Montreal Children's Hospital Research Ethics Board. Informed consent was obtained for all genetic analyses from the parents of this boy and for publication of this case report.

## Discussion

XLAG often manifests in the first year of life and is more prevalent in girls. Unlike our case, all previously reported patients have had either sporadic or familial germline Xq26.3 microduplications allowing a definitive diagnosis on leukocyte-derived DNA. Because our case presented with a typical phenotype, we tested the pituitary tissue sample, and this showed *GPR101* microduplication. One of the four different genomic DNA samples we tested from various tissues also showed *GPR101* duplication, suggesting that this patient has somatic mosaicism as a result of a postzygotic mutation. No cases of somatic mosaicism have been described previously in patients with XLAG syndrome.

Treatment of XLAG is challenging. Previously described cases treated with surgery followed by medical treatment and/or radiotherapy have often developed hypopituitarism and central diabetes insipidus (1–3, 6). Management of our patient with cabergoline, continuous sc infusion of short-acting somatostatin analog, and pegvisomant proved successful in controlling growth without hypopituitarism or other significant side effects.

## Conclusion

This is the first report on a patient with documented somatic *GPR101* duplication leading to XLAG. The case is instructive because it: 1) demonstrates that a negative genetic test for Xq26.3 microduplication or *GPR101* duplication on peripheral blood DNA does not exclude the diagnosis of XLAG because it can result from a somatic mutation affecting the pituitary and possibly other tissues as a result of somatic mosaicism; and 2) proposes an effective combination and route of medical treatment for this condition that can be acceptable for young children.

## References

[1] Trivellin G Daly AF Faucz FR Gigantism and acromegaly due to Xq26 microduplications and GPR101 mutation. N Engl J Med. 2014;371:2363–2374. 25470569 10.1056/NEJMoa1408028 PMC4291174

[2] Beckers A Lodish MB Trivellin G X-linked acrogigantism syndrome: clinical profile and therapeutic responses. Endocr Relat Cancer. 2015;22:353–367. 25712922 10.1530/ERC-15-0038 PMC4433400

[3] Rostomyan L Daly AF Petrossians P Clinical and genetic characterization of pituitary gigantism: an international collaborative study in 208 patients. Endocr Relat Cancer. 2015;22:745–757. 26187128 10.1530/ERC-15-0320 PMC6533620

[4] Canadian Paediatric Society. WHO Growth Charts. Promoting optimal monitoring of child growth in Canada: using the WHO growth charts. http://www.cps.ca/tools-outils/who-growth-charts Accessed December 27, 2015.

[5] Näntö-Salonen K Koskinen P Sonninen P Toppari J Suppression of GH secretion in pituitary gigantism by continuous subcutaneous octreotide infusion in a pubertal boy. Acta Paediatr. 1999;88:29–33. 10090543 10.1111/j.1651-2227.1999.tb01263.x

[6] Naves LA Daly AF Dias LA Aggressive tumor growth and clinical evolution in a patient with X-linked acro-gigantism syndrome. Endocrine. 2016;51:236–244. 26607152 10.1007/s12020-015-0804-6 PMC5497487

